# Sleep medications and alcohol abuse masked sleep apnea syndrome without sleepiness for over 30 years: A case report

**DOI:** 10.1002/pcn5.70119

**Published:** 2025-05-07

**Authors:** Naoya Onishi, Keiichiro Nishida, Hironari Minami, Katsunori Toyoda, Yoshitaka Nishizawa, Yoichiro Kubo, Soichiro Ikeda, Tetsufumi Kanazawa

**Affiliations:** ^1^ Department of Neuropsychiatry Osaka Medical and Pharmaceutical University Osaka Japan; ^2^ Division of Respiratory Medicine and Thoracic Oncology, Department of Internal Medicine (I) Osaka Medical and Pharmaceutical University Osaka Japan

**Keywords:** alcohol abuse, benzodiazepines, frontal disfunction, insomnia, sleep apnea syndrome

## Abstract

**Background:**

Insomnia is often treated with benzodiazepines, which can lead to long‐term use and potential complications, especially if underlying conditions like sleep apnea syndrome (SAS) go undiagnosed. This case report describes a 70‐year‐old woman with chronic insomnia, benzodiazepine and alcohol abuse, and a previously undiagnosed severe SAS.

**Case Presentation:**

The patient, a 70‐year‐old woman was admitted for suspected delirium caused by benzodiazepine and alcohol abuse. She had been prescribed benzodiazepines by multiple health care providers for over 30 years. Additionally, she regularly consumed alcohol to self‐medicate. These were replaced and treated with diazepam, but her sleep disturbances persisted. She had a noticeable snoring during her hospitalization, which led to a polysomnography. The results revealed that she had severe SAS with an apnea–hypopnea index of 57.1 during total sleep time. Continuous positive airway pressure therapy significantly improved her sleep quality. She also underwent testing for dementia, which showed frontal lobe dysfunction on imaging and psychological testing. She was discharged after 66 days of treatment, able to live independently with home care support.

**Conclusion:**

This oversight led to prolonged use of benzodiazepines and self‐medication with alcohol, worsening her condition. The case emphasizes the need for thorough assessments in patients with treatment‐resistant insomnia, particularly to identify organic causes like SAS that may be contributing to their symptoms. This case highlights the importance of reevaluating patients whose insomnia does not improve with sleep medication.

## BACKGROUND

Insomnia is a common disorder in modern society. It is linked not only to long‐term mental health issues but also to chronic physical conditions, such as diabetes and hypertension, making its treatment crucial for quality of life. Generally, insomnia is more common in older adults than in younger individuals, with over 50% of older individuals experiencing difficulty initiating sleep or waking at night.^1^ Initial treatment options include lifestyle modifications, such as establishing a regular daily routine. Benzodiazepines have often been used to treat insomnia.

One study indicated that approximately 5.2% of American adults aged 18–80 years utilized benzodiazepines, with the prevalence of use increasing with age.^2^ A Japanese survey of 74,044 outpatients newly prescribed benzodiazepines reported that 6687 (9%) had been prescribed for more than 8 months^3^ and long‐term use of benzodiazepines is particularly problematic in Japan.^4^

In addition to pharmacotherapy, some individuals may use alcohol as self‐medication. However, alcohol consumption markedly worsens sleep quality and exacerbates insomnia. Mixing benzodiazepines and alcohol can lead to excessive sedation, cognitive and psychomotor impairments, impulsive behavior, and other symptoms owing to their combined depressive effects.

Sleep apnea syndrome (SAS) is a leading cause of insomnia and is diagnosed using polysomnography, with an apnea–hypopnea index (AHI) of five or more events per hour. An AHI score of 30 or higher is diagnosed as severe SAS. Without appropriate treatment of SAS, the use of substances such as benzodiazepines and alcohol can aggravate the condition. In some instances, sleep medications are prescribed without a proper SAS diagnosis in psychiatric settings.

This study presents the case of an older woman admitted to hospital for suspected delirium resulting from the abuse of benzodiazepines and alcohol. Throughout her inpatient treatment, we identified a previously undiagnosed SAS originating in her youth. Written informed consent was obtained from the patient for the publication of this case report and any accompanying images.

## CASE PRESENTATION

The patient, a 70‐year‐old woman, had a problem‐free childhood with her parents and became a cook. After marrying her husband, she had four children. As her children grew up, she experienced increased levels of depression. Living alone with her husband, she was intermittently treated for persistent depressive disorder with paroxetine in year X‐30, along with lipid‐lowering drugs and sleep medications prescribed by her primary care physician. These medications allowed the patient to manage her household and enjoy socializing and playing the violin. Following her husband's death from year X, she exhibited heightened anxiety, depressive mood, worsening insomnia, reduced motivation, frequent day and night calls to her children, and disorganized speech. Consequently, the patient was referred to our psychiatric department by her family physician.

**Figure 1:**
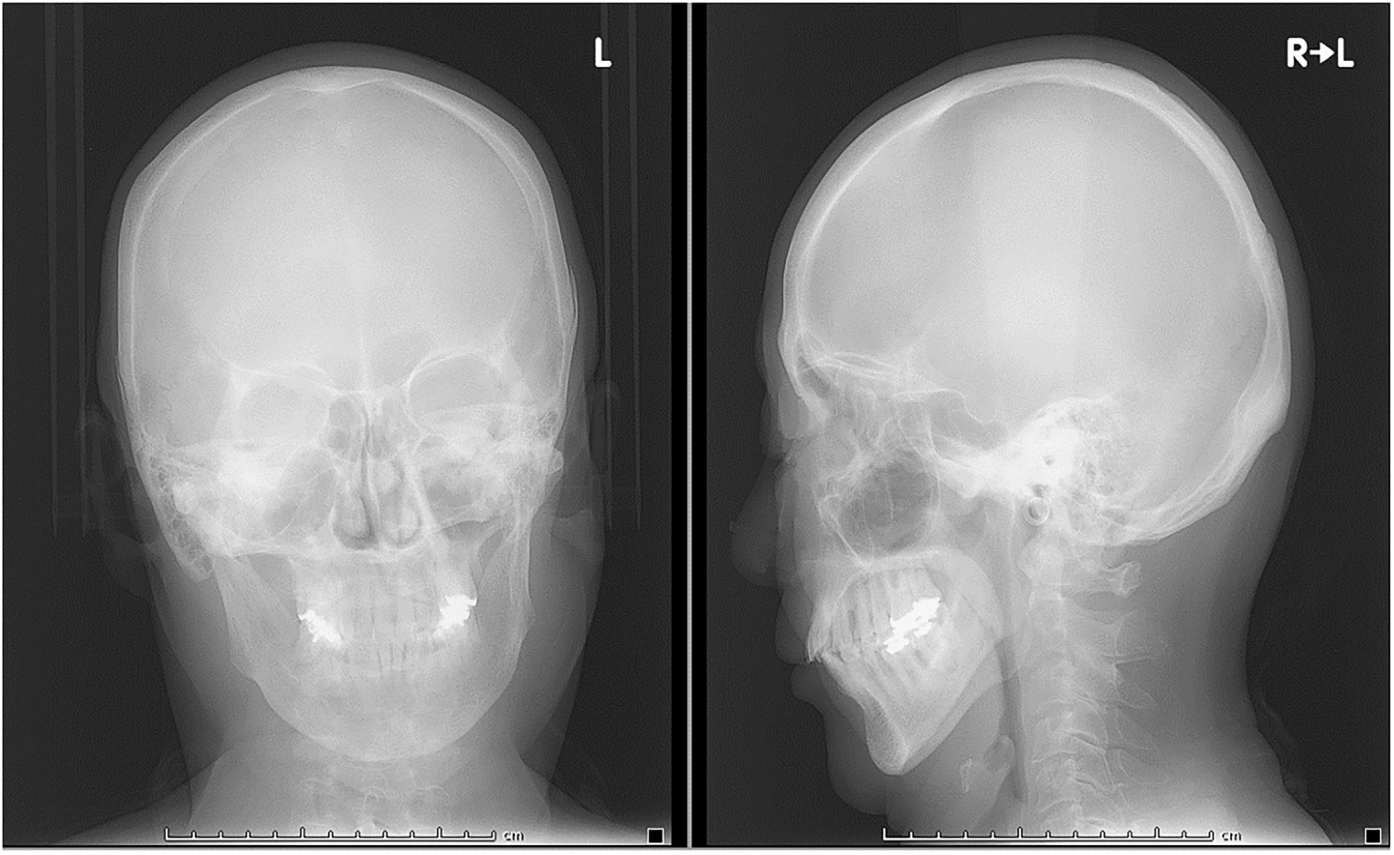
Nasal X‐ray showing the nasal septum is shifted toward the right nasal cavity.

**Figure 2:**
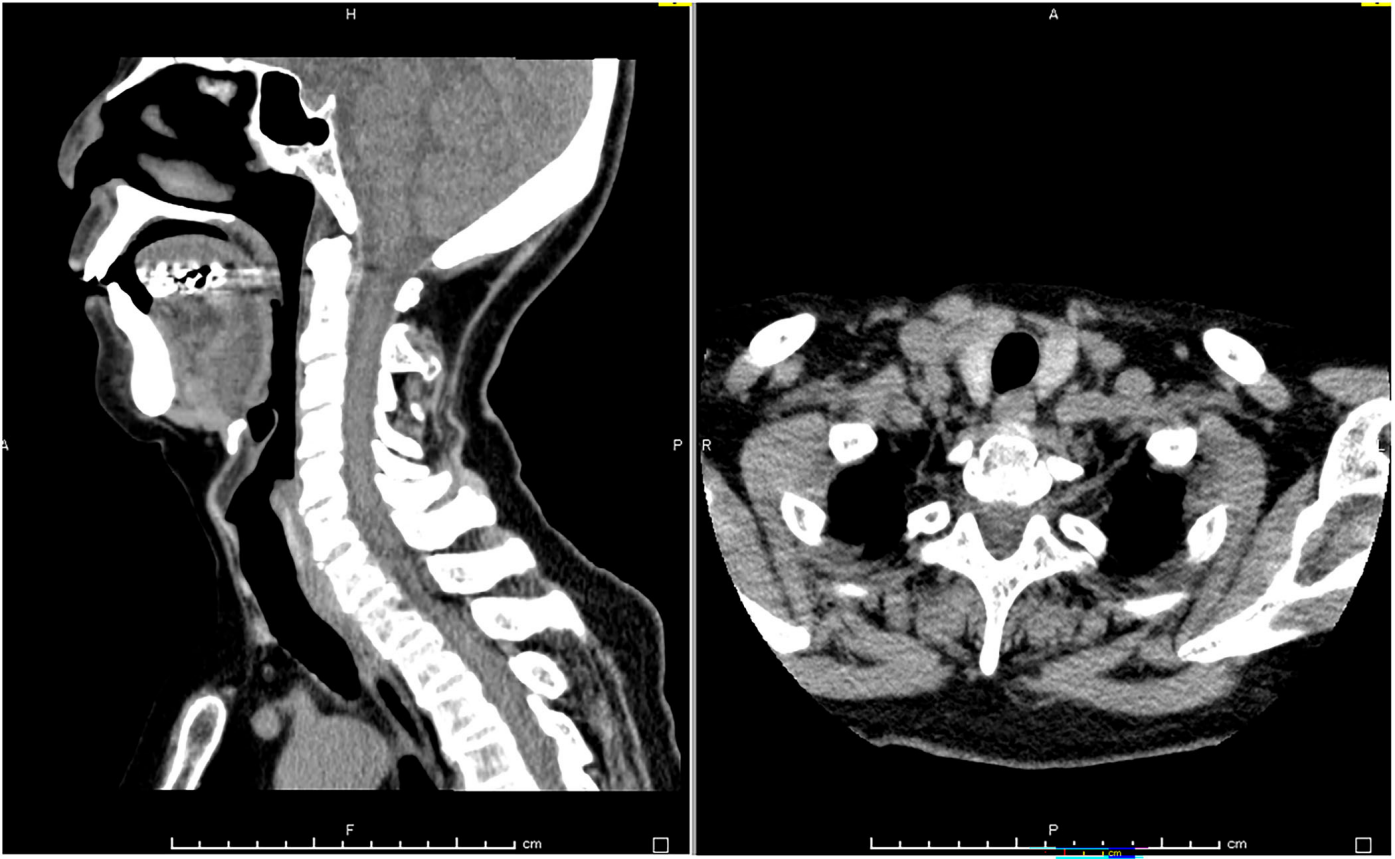
The computed tomography scan showed no tumor or other organic disease causing the snoring.

On assessment, she and her family sought treatment for anxiety and depression, which led to her hospitalization. She struggled to initiate sleep and experienced mid‐sleep awakenings, seeking deeper sleep than that provided by her current medications. For the past 10 years, she had been prescribed high doses of benzodiazepines, including brotizolam, etizolam, nitrazepam, roflazepam, and zolpidem (a total of 14.66 diazepam equivalents), from four different medical facilities without coordination. She consumed benzodiazepines mixed with 100 mL of sake (a Japanese alcoholic beverage) every 2 h from midnight to 10 a.m. (net alcohol content 72 g). To manage withdrawal symptoms from benzodiazepine and alcohol abuse, 20 mg of diazepam was introduced on the first day of admission to prevent withdrawal, and lenvorexant and quetiapine were administered. The dose of diazepam was gradually reduced and discontinued by the 23rd day. Her anxiety, depressive mood, and lack of motivation improved with environmental changes and the ongoing use of paroxetine (25 mg). However, she experienced loud snoring and mid‐sleep awakenings—symptoms she reported having since adolescence but had never sought treatment for. Notably, there were no complaints of daytime sleepiness, which was rated as 0 on the Japanese version of the Epworth Sleepiness Scale (JESS). On the 23rd day, a consultation with a respiratory physician was conducted due to the suspicion of SAS, and polysomnography was subsequently performed. The results revealed a sleep latency of 4.5 min, a REM latency of 175.5 min, and a sleep efficiency of 76.3%. An abnormal distribution of sleep stages was also observed: 11.9% in REM sleep (Stage R), 56.7% in Stage N1, 31.4% in Stage N2, and 0% in Stage N3. The AHI was 26.2/h during REM sleep, 61.2/h during NREM sleep, and 57.1/h (obstructive apnea 86% and hypopnea 14%) for total sleep time. The mean oxygen saturation was 93%, with a minimum of 83%. The periodic limb movement index (PLMI) was 0.7/h and the arousal index was 50.5/h. X‐rays (Figure 1) and computed tomography (CT) scans (Figure 2) of her face and head revealed micrognathia (receding and underdeveloped lower jaw), with no evidence of tumors or other organic diseases. Based on these findings, a diagnosis of severe sleep apnea syndrome was established.

On day 28, continuous positive airway pressure (CPAP) therapy was initiated, resulting in an improvement in the AHI to 3.9 events per hour. Additionally, loud snoring, nocturia (defined as urination twice per night), and chronic migraine resolved.

**Figure 3:**
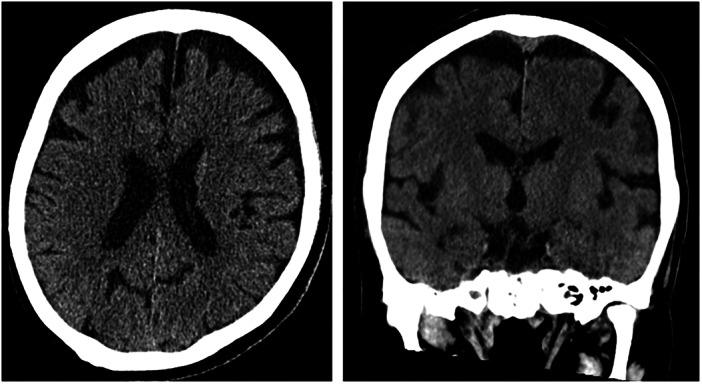
Computed tomography of the head on the 29th day in the hospital showing preserved hippocampal volume but significant atrophy of the frontal lobe.

**Figure 4:**
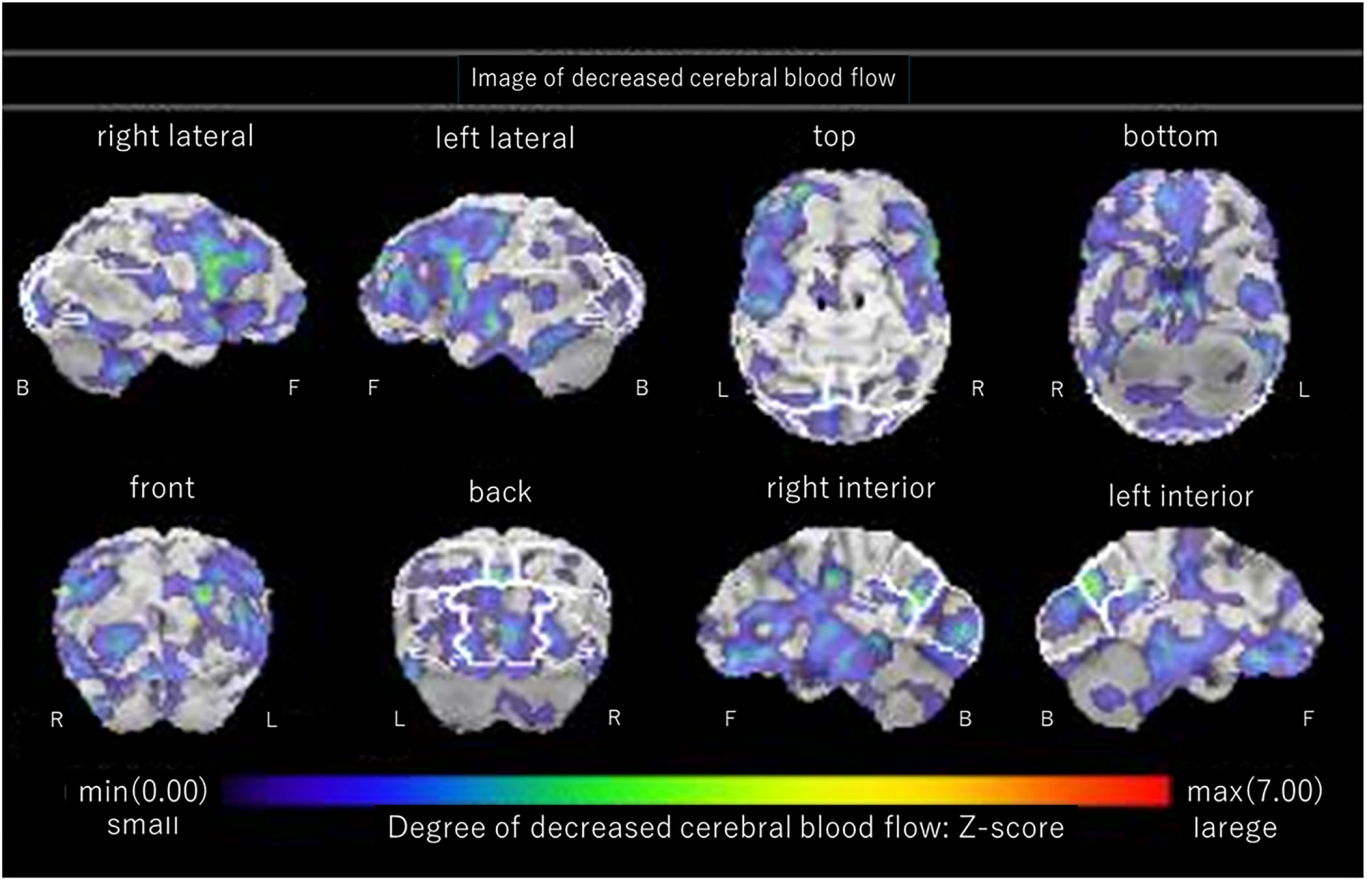
Single photon emission computed tomography on the 42nd day in the hospital indicating indicated reduced blood flow in both frontal lobes.

Furthermore, the possibility that dementia may have contributed to these symptoms was considered, therefore the patient underwent CT on day 29 (Figure 3) and single‐photon emission CT (SPECT) on day 42 (Figure 4). The CT revealed atrophy in the frontal lobes and SPECT indicated reduced blood flow in the same areas. She scored Mini‐Mental State Examination score of 26/30, Montreal Cognitive Assessment (MoCA‐J) 24/30, Frontal Assessment Battery score (FAB) of 13/18, and Behavioral Assessment of the Dysexecutive Syndrome (BADS) score 89. Considering her condition during the latter half of her stay, she was able to live independently at home with home nursing care and was discharged on the 66th day.

One year after discharge from the hospital, the HAMD score was 2, indicating a significant improvement in depressive symptoms and the attainment of remission status.

The patient had been consistently attending outpatient visits in a stable condition, and reports from home‐visit nursing services indicate an increased level of independence compared to previous assessments. Furthermore, no evidence of alcohol or sedative misuse had been observed. Cognitive function also demonstrated improvement, with scores of 15/18 on the FAB and 114 on the BADS. The MoCA‐J score was 22/30, suggesting minimal cognitive decline.

## DISCUSSION AND CONCLUSION

The anxiety symptoms in this case were likely associated with the inappropriate use of benzodiazepines and alcohol. In the early stages of hospitalization, these symptoms were influenced by withdrawal from these substances but resolved relatively quickly with medication adjustments based on gradual tapering and the stability of the hospital environment. Immediately after ingesting benzodiazepines and alcohol, the patient experienced a temporary reduction in anxiety. However, within a few hours, she recognized that her anxiety had actually worsened, allowing her to reflect on her past substance misuse. On the other hand, we consider sleep improvement to be the most critical factor in alleviating her depressed mood and lack of motivation in the long‐term. Initially, the patient was not fully aware that her mood improvements were linked to better sleep. However, after starting SAS treatment, she noticed declining daytime fatigue and sleepiness, which gradually led to improved mood and increased motivation. Finaly, a complete resolution of symptoms was observed.

The patient had been snoring since adolescence but was unaware that SAS was contributing to her insomnia and therefore did not report it during medical consultations. According to the International Classification of Sleep Disorders, approximately 60% of moderate to severe SAS cases are estimated to be attributable to obesity. On the other hand, SAS patients with normal or below‐normal weight are more prone to upper airway obstruction due to local morphological abnormalities such as upper and lower jaw dysplasia and tonsillar hypertrophy. Postmenopausal women are also considered to have an increased risk of obstructive sleep apnea (OSA). In this case, a woman with a small jaw and no obesity is thought to have experienced worsening obstructive apnea approximately 30 years ago when she underwent menopause. However, neither she nor her healthcare providers recognized the complications of SAS due to the widespread belief that obesity is the predominant cause of the condition. Consequently, these factors made the diagnosis of SAS challenging, leading to a delay of more than 30 years before an accurate diagnosis was established and benzodiazepines were prescribed. Furthermore, inadequate information sharing among the patient, family members, and healthcare providers may have contributed to the prolonged diagnostic delay.

Moreover, she consumed alcohol to aid sleep, as suggested by her husband. These instances of substance misuse led to increased daytime sleepiness; however, they did not contribute to the treatment of SAS and were instead counterproductive. The patient remained unaware that SAS was the cause of her insomnia until polysomnography and other diagnostic tests were conducted during this hospitalization.

Although polysomnography is the examination with the highest level of evidence and is adopted in the diagnostic criteria for SAS, it is not feasible to perform polysomnography on all patients with insomnia. However, studies have reported that approximately one‐third of Japanese SAS patients are not obese, and the misconception that the absence of obesity rules out SAS can be a significant factor in missed diagnoses, particularly in Asian populations. Clinicians should therefore inquire about symptoms suggestive of SAS, with particular attention to snoring. Characteristic features of SAS‐related snoring include episodes of breathlessness, nasal breathing, witnessed apneas, and silent periods following loud snoring. These specific snoring patterns serve as valuable clinical indicators for diagnosing SAS.

SAS is a common cause of insomnia. Adam et al. reported that approximately 1 billion adults aged 30–69 years globally may have SAS, with an estimated 425 million suffering from moderate‐to‐severe obstructive sleep apnea, for which treatment is typically recommended.^5^ Despite this, benzodiazepines are frequently prescribed to patients with insomnia, often without a thorough examination for SAS, leading to a situation where sleep medication is prescribed without addressing the underlying cause.

This patient did not report daytime sleepiness. Her score on the JESS was 0, indicating an absence of sleepiness during daytime.

Regarding the differential diagnosis of dementia, CT and SPECT revealed atrophy of the frontal lobe and a corresponding decrease in blood flow, raising the possibility of early‐stage dementia. However, based on the results of the MoCA‐J and the FAB as well as clinical symptoms, dementia was considered unlikely. Continuous monitoring is necessary to determine whether the brain atrophy is a transient effect of alcohol and benzodiazepine abuse or an early sign of a neurodegenerative disease.

This case study suggests that patients with chronic insomnia should be evaluated for the presence of SAS and other disorders, as well as for brain function. The muscle relaxant effects of benzodiazepines and alcohol worsen SAS, inducing delirium and creating a vicious cycle. A thorough examination of organic diseases related to sleep disorders is essential in clinical practice. However, many patients are prescribed sleep medications without adequate investigation. This case highlights the importance of reevaluating patients whose insomnia does not improve with sleep medication, as underlying organic conditions may be present.

## AUTHOR CONTRIBUTIONS

Naoya Onishi and Keiichiro Nishida drafted the manuscript. Tetsufumi Kanazawa contributed to review and editing. Hironari Minami, Katsunori Toyoda, Yoshitaka Nishizawa, and Yoichiro Kubo managed diagnosis and treatment during hospitalization and reviewed the manuscript. Soichiro Ikeda conducted detailed evaluation and treatment for the patient's sleep apnea syndrome. All the authors have read and approved the final manuscript.

## CONFLICT OF INTEREST STATEMENT

The authors declare no conflicts of interest.

### ETHICS APPROVAL STATEMENT

We obtained approval from the Ethics Committee of Osaka Medical and Pharmaceutical University.

## PATIENT CONSENT STATEMENT

Consent was obtained from the patient prior to the case report.

## CLINICAL TRIAL REGISTRATION

N/A.

## Data Availability

N/A.
